# Blended electrospinning with human liver extracellular matrix for engineering new hepatic microenvironments

**DOI:** 10.1038/s41598-019-42627-7

**Published:** 2019-04-18

**Authors:** Rhiannon Grant, John Hallett, Stuart Forbes, David Hay, Anthony Callanan

**Affiliations:** 1 Institute for Bioengineering, School of Engineering, University of Edinburgh, Scotland, UK; 2 Scottish Centre for Regenerative Medicine, University of Edinburgh, Scotland, UK

**Keywords:** Biomaterials - cells, Biomaterials - proteins, Tissues, Tissue engineering

## Abstract

Tissue engineering of a transplantable liver could provide an alternative to donor livers for transplant, solving the problem of escalating donor shortages. One of the challenges for tissue engineers is the extracellular matrix (ECM); a finely controlled *in vivo* niche which supports hepatocytes. Polymers and decellularized tissue scaffolds each provide some of the necessary biological cues for hepatocytes, however, neither alone has proved sufficient. Enhancing microenvironments using bioactive molecules allows researchers to create more appropriate niches for hepatocytes. We combined decellularized human liver tissue with electrospun polymers to produce a niche for hepatocytes and compared the human liver ECM to its individual components; Collagen I, Laminin-521 and Fibronectin. The resulting scaffolds were validated using THLE-3 hepatocytes. Immunohistochemistry confirmed retention of proteins in the scaffolds. Mechanical testing demonstrated significant increases in the Young’s Modulus of the decellularized ECM scaffold; providing significantly stiffer environments for hepatocytes. Each scaffold maintained hepatocyte growth, albumin production and influenced expression of key hepatic genes, with the decellularized ECM scaffolds exerting an influence which is not recapitulated by individual ECM components. Blended protein:polymer scaffolds provide a viable, translatable niche for hepatocytes and offers a solution to current obstacles in disease modelling and liver tissue engineering.

## Introduction

According to the NHS, liver disease is one of the top five causes of premature death in the UK, with incidence rising sharply by 20% over the last decade^1^. While liver disease incidence is rising, other top healthcare burdens, such as stroke, cancer, heart disease and lung disease mortality rates continue to fall^2,3^.

Liver disease’s hallmark pathology of late diagnosis and rapid acute disease progression leads to an urgent need for donor organs; the only curative treatment for end stage liver disease^4^. However, a chronic and ongoing shortage of suitable organs for transplant means many die before a donor liver can be found, and countless others live with severe, debilitating symptoms at a high cost to both the patient and the healthcare system^2^.

As part of the push for a solution to this problem, tissue engineers are focussing on creating niche microenvironments for main cell type of the liver, the hepatocyte, which support cell survival and function and could be used to treat patients in the future^5–9^. Such an environment would also allow for the study of new pharmaceuticals to treat human disease more effectively^10^. While research to date is making inroads into this dilemma, we are yet to see a lab created environment which accurately recapitulates the complex, finely tuned and responsive extracellular matrix (ECM) of the liver^11,12^.

In an effort to address this, researchers have been incorporating bioactivity into scaffold environments for hepatocytes^6,7,11^. Decellularized extracellular matrix is the obvious avenue for such research^13,14^, and while results are promising a decellularized liver still requires a donor liver and recellularization. Obstacles such as necrosis, immune reaction and residual decellularization agents are all yet to be fully addressed for decellularized whole organs to be a truly viable option^14–17^. Individual ECM components in the form of gelatin^18,19^, collagen^20–22^, laminin^23–25^ and fibronectin^26–28^ have all been employed; each influencing the hepatocytes survival and function and providing insight into the complex cell-matrix interactions present in the hepatic microenvironment. However each protein individually represents a small fraction of the bioactive molecules present in the ECM and when used in isolation cannot recapitulate the healthy hepatic matrix^24,29,30^.

With this work in mind we created a new scaffold for liver tissue engineering; for the first time incorporating human liver ECM (hLECM) directly into the fibres of electrospun polymer scaffolds. By combining the best of current scaffolding technology; reproducible polymeric scaffolds and efficiently decellularized human donor liver we have created a niche bioinfluential microenvironment which influences the function of cultured human hepatocytes.

## Results

Our results demonstrate a method of incorporating proteins directly into a scaffold environment and of making impactful use of a valuable tissue resource which would otherwise be wasted. Protein:polymer scaffolds containing human liver ECM exert a significant positive influence on the gene expression profile, albumin production, attachment, and survival of liver cells which cannot be recapitulated by individual ECM components. These scaffolds show great potential not only for the future of liver tissue engineering and patient treatment, and are easily adaptable for other organs and tissues.

### Mechanical characterization of scaffolds

**Figure 1:**
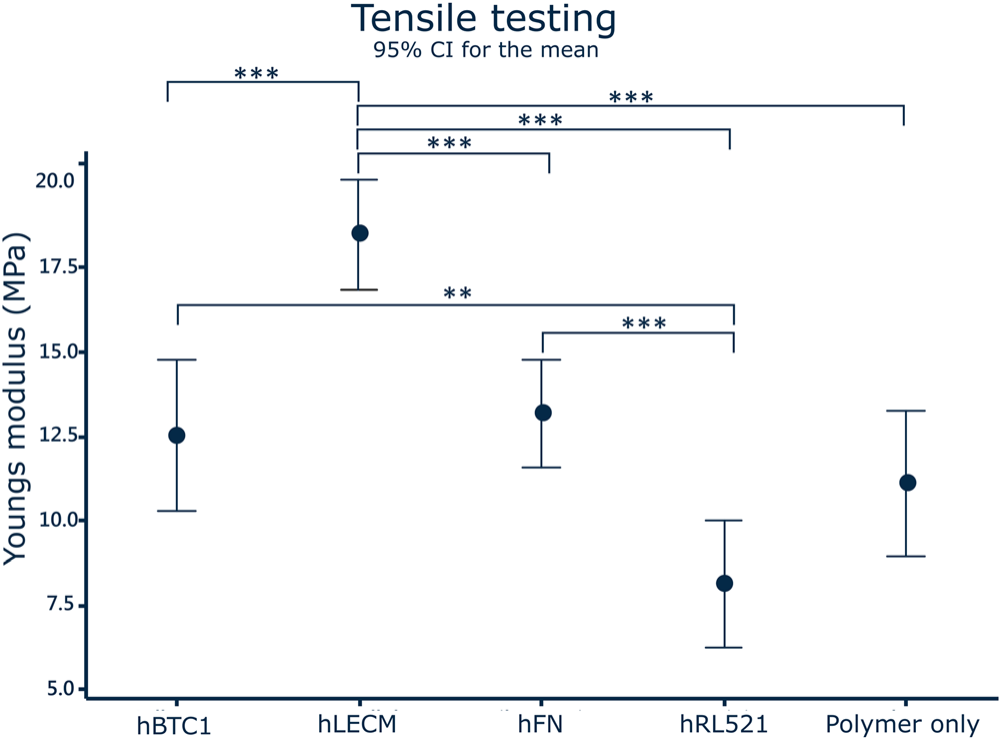
Mechanical testing. Incorporating human liver ECM into the scaffold produces a significantly stiffer environment for hepatocytes. hRL521 scaffolds are significantly more elastic than both hBTC1 and hFN scaffolds. N = 6, Data shown as mean ± 95% confidence interval with statistics performed using One way ANOVA with Games Howell post hoc analyses.

Tensile testing revealed significant increases in the Young’s Modulus between the ECM scaffold and every other scaffold (Fig. 1), indicating that incorporating human liver ECM into the scaffold results in a significantly stiffer environment for hepatocytes. Interestingly, hRL521 scaffolds were significantly more elastic than both hBTC1 and hFN scaffolds (Table 1). Such results demonstrate that the mechanical influence of varying ECM compositions influence on cell behaviour and function cannot be discounted in studies and must be considered when analysing biological results.

**Table 1:** Elasticity (0–10% strain).

Scaffold	Polymer only	hBTC1	hFN	hRL521	ECM
Average Young’s Modulus (MPa)	11.11 ± 1.88	12.53 ± 1.96	13.2 ± 1.40	8.15 ± 1.35	18.49 ± 1.40

### Histological characterisation of the scaffolds

**Figure 2:**
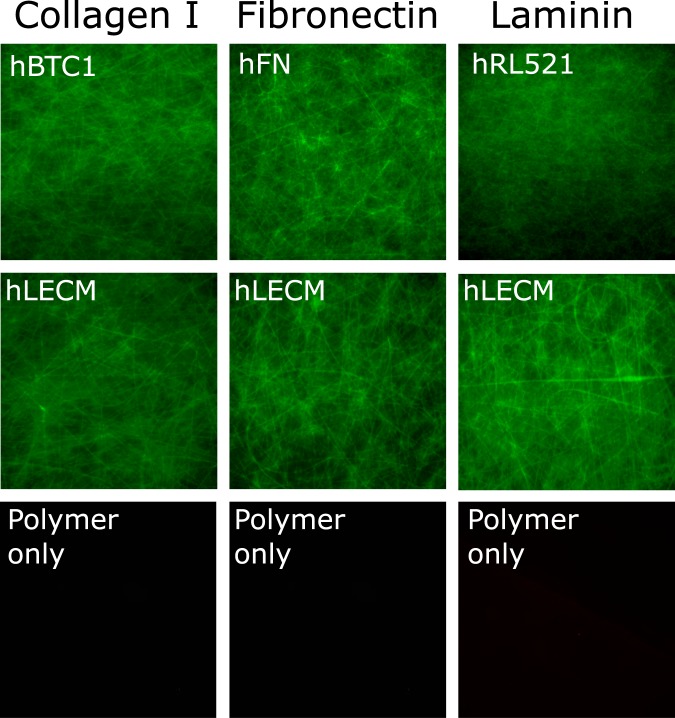
Immunohistochemistry. Collagen I, Fibronectin and Laminin are all present in their respective single protein scaffolds, as well as in varying degrees in the ECM scaffold, with no false positive staining observed in the control polymer only condition. Stains were performed for Collagen I, Laminin and Fibronectin,post processed using ImageJ. 10x magnification.

The presence of the various proteins was demonstrated by immunohistochemistry performed on the scaffolds (Fig. 2). Collagen I, Laminin and Fibronectin were chosen because of their long established influence on hepatic cells^29,31,32^. Fibronectin is ubiquitous in healthy liver, collagen I is the largest component of the healthy liver extracellular matrix and laminin is of particular importance in the differentiation of liver cells as well as for cell adhesion and liver regeneration^11,33^. Each is clearly present in their respective single protein scaffolds, as well as in varying degrees in the ECM scaffold, with no false positive staining observed in the control polymer only condition. Reassuringly, this indicates that the antigens to which the primary antibodies bind were not affected by the solubilisation or electrospinning process, and therefore that some bioactivity is maintained throughout the scaffold fabrication process.

### Cell attachment and survival on scaffolds

**Figure 3:**
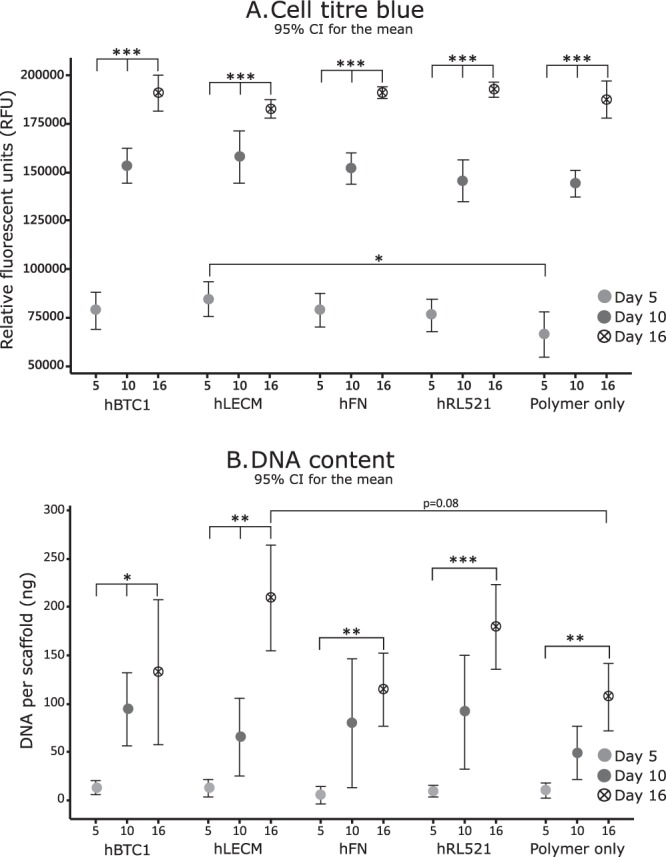
Cell viability – Cell titre blue and picogreen DNA quantitation. Cell adherence was assessed by CellTiter-Blue® Cell viability assay (**A**) and further confirmed by Quant-IT™ Picogreen® dsDNA assay (**B**). Minimum n = 5. Data shown as mean ± 95% confidence interval with statistics performed using One-way ANOVA with Tukey post hoc testing. *p < 0.05 **p < 0.01, ***p < 0.001. Each condition maintains cell survival and the number of cells increases significantly between each time point (**A**). A significant increase in fluorescence is observed between the polymer only and hLECM scaffolds at day 5 indicating that the presence of human liver ECM has a positive influence on the early expansion and/or survival of hepatocytes. A consistent pattern is observed in the DNA concentration (**B**) on the scaffolds.

**Figure 4:**
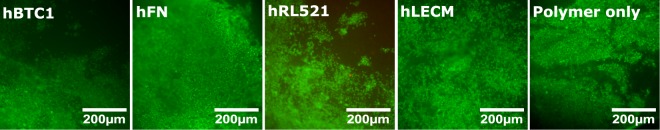
Cell viability – Live Dead. Live/Dead® Viability/Cytotoxicity staining analyses demonstrates continuing survival of the hepatocytes at the latest time point (16d), with a confluent population of viable cells and a low level of cell death on each scaffold. Results demonstrate the FL is viable at all assessed time points. 10x magnification.

**Figure 5:**
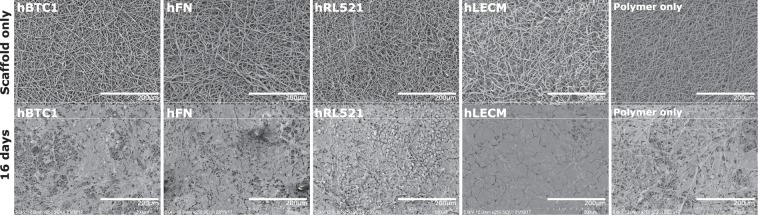
SEM characterization. The scaffolds were assessed for consistency and fibre size via scanning electron microscopy and subsequent image analysis. Fibres diameter was determined by DiameterJ 34, n = 4. 250x magnification. Presence of a dense cell layer is further confirmed by SEM imaging with a carpet of cells visible in each condition at 16d.

Every scaffold maintains the survival of the THLE-3 liver cells and the number of cells increases significantly between each time point (Fig. 3A). Interestingly, between conditions the only significant difference observed is between the polymer only and hLECM scaffolds at day 5, with higher fluorescence observed on the hLECM scaffolds indicating that the presence of human liver ECM in the scaffold has a positive influence on the early expansion and/or survival and adherence of hepatocytes. Reassuringly, a similar pattern is observed in the DNA concentration (Fig. 3B) on the scaffolds, with a difference observed between polymer only and hLECM conditions once again; more DNA is present on the hLECM scaffolds indicating the presence of more cells. Live/dead viability/cytotoxicity images (Fig. 4) further demonstrate the continuing survival of the hepatocytes at the latest time point (16d), with a confluent population of viable cells present on the scaffold and a low level of cell death on each scaffold. Presence of a dense cell layer is further confirmed by SEM imaging (Fig. 5), with a carpet of cells visible in each condition at 16d.

### Gene expression of THLE-3s in response to hybrid polymer-ECM scaffolds

**Figure 6:**
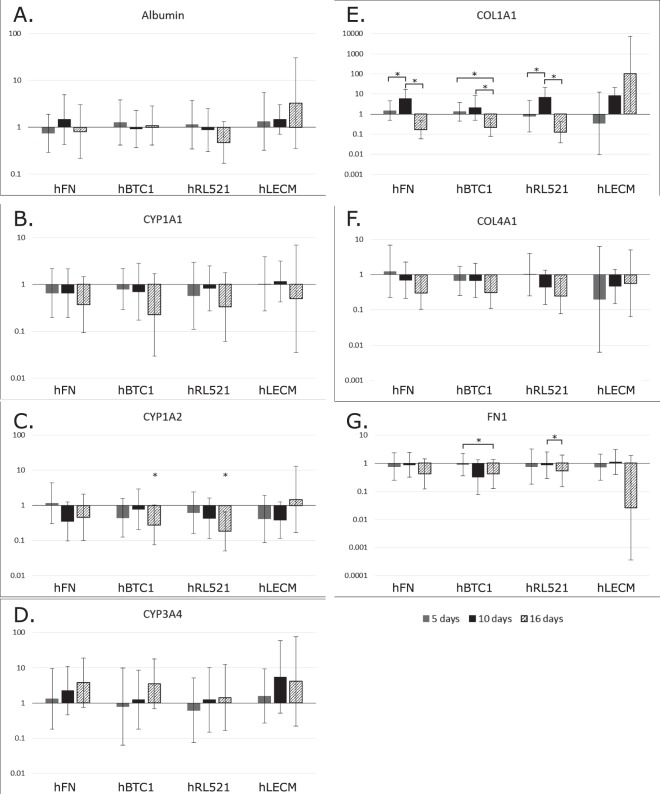
Q-PCR of key hepatic genes. Quantitative analysis of gene expression was undertaken on the functional cell layer at five, ten and sixteen days of culture, compared to that of the same culture periods grown on polymer only scaffolds. mRNA levels of Albumin (**A**), Cyp1A1 (**B**), Cyp1A2 (**C**), CYP3A4 (**D**), Collagen I (**E**), and Collagen IV (**F**) and Fibronectin (**G**) are represented as fold difference relative to polymer only controls and relative to the housekeeping gene GAPDH. One-way ANOVA with Games Howell and Tukey post hoc testing and minimum n = 5. *p < 0.05 **p < 0.01, ***p < 0.001. Error bars = SD.

Genes associated with liver function were assayed for gene expression (Fig. 6). Additionally, genes for ECM expression were assayed. Albumin (Fig. 6A), a marker of appropriate adult hepatocyte differentiation and function, and Cytochrome P450s (CYP1A1 Fig. 6B, CYP1A2 Fig. 6C and CYP3A4 Fig. 6D), a family of enzymes involved in metabolism of drugs and other toxic compounds in the liver^34–36^ were both studied. Three ECM genes important in normal liver composition were also assayed^24,33^; Fibronectin (FN1 Fig. 6E), Collagen I (COL1A1 Fig. 6F) and Collagen IV (COL4A1 Fig. 6G). Considering the plastic nature of ECM, these genes are of interest with regards to ongoing modification of the hepatic niche despite hepatocytes not being the sole producers of hepatic ECM^33,37^. Finally, expression of alpha-1 antitrypsin (AAT Supplementary Fig. 1A) and hepatocyte nuclear factor 4 (HNF Supplementary Fig. 1B) were tested. AAT is a serpin family protease inhibitor which protects tissues frim adverse effects of inflammatory enzymes released during immune responses^38^. HNF binds DNA and controls the expression of several hepatic genes^39^. Both genes are considered as markers of hepatic differentiation. Results were normalised to the polymer only condition to assay the influence of the protein component of the scaffolds on gene expression.

Only the human liver ECM scaffold maintains albumin expression in the expected pattern, increasing over time; as seen in primary hepatocytes and other liver cell lines^40^. CYP1A1 is consistently downregulated in comparison to the polymer only scaffolds in every condition over the 16 days. CYP1A2 is down regulated in comparison to polymer only, with significant changes observed on hRL521 and hBTC1 scaffolds at day 16. CYP1A2 is upregulated at day 16 on liver ECM, indicating an improvement in metabolic capability for hepatocytes grown in the hLECM scaffolds. All the single protein environments increase the expression of CYP3A4 in comparison to the polymer only environment. These changes in metabolic genes demonstrate the importance of protein microenvironment for hepatocyte function. hLECM scaffolds caused an increase in COL1A1 expression over the 16 days, with expression 100 times higher at 16 days than on the polymer only environment. Significant changes were observed between days 5 and 10, and 10 and 16 of hFN and hRL521 scaffolds, and between days 5 and 16, and 10 and 16 on hBTC1 scaffolds. COL4A1 was downregulated over time on all the single protein scaffolds, but increases over time on the scaffolds which incorporate hLECM, although is overall reduced in comparison to polymer only. Significant changes were observed between days 5 and 16 on hBTC1 and days 10 and 16 on hRL521 scaffolds in FN1 expression. Reduction in expression of FN1 was slight in comparison to polymer only scaffolds. HNF and AAT expression are low as expected^41^ (Supplementary Fig. 1), however the relatively short culture period of 16d may not yet have revealed whether or not these cells are capable of mature hepatic gene expression.

### Albumin production

**Figure 7:**
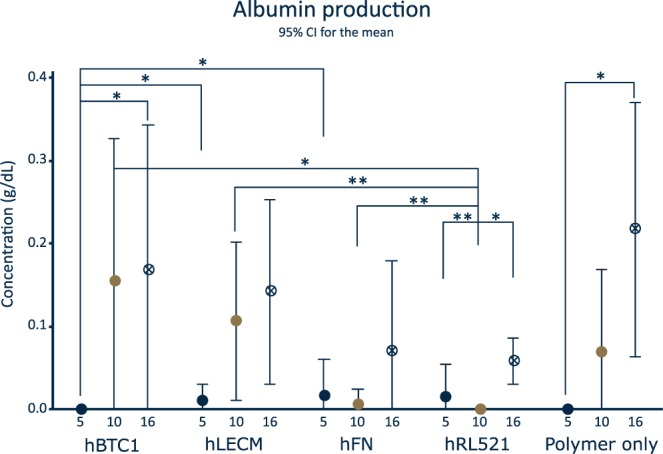
Albumin production Serum. albumin produced by the THLE-3 cells over 24 hours at 5, 10 and 16 day timepoints. n = 6. Data shown as mean ± 95% confidence interval with statistics performed using One-way ANOVA with Games Howell and Tukey post hoc testing and minimum n = 5. *p < 0.05 **p < 0.01, ***p < 0.001.

Albumin levels are indicative of hepatic health *in vivo* and response to the cellular microenvironment^7,29^. hBTC1, polymer only and liver ECM all exhibit increasing production of albumin over time (Fig. 7), as expected in a healthy hepatocyte culture and correlating with gene expression patterns observed in Fig. 6. Interestingly, a significant increase in albumin production is observed on liver ECM scaffolds when compared with hRL521 scaffolds at day 10, and compared to hBTC1 scaffolds at day 5; indicating that hLECM is important for the production of albumin and that individual ECM components are not sufficient to boost the production of albumin.

### Validation of tissue decellularization

Picogreen was employed to validate the decellularization process and ensure minimal DNA was present in the scaffolds. Results in Table 2 reassure us that our decellularization process is effective.

**Table 2:** Remnant DNA in scaffolds.

Scaffold	Native liver	Decellularized liver	Polymer only	hBTC1	hFN	hRL521	hLECM
DNA (ng p/mg)	340.57	0	0	0	0	0	0

## Discussion

A bioactive scaffold for hepatocyte culture is an important avenue for tissue engineering, meeting the need for an environment which supports the behaviour and function of hepatocytes in as close to an *in vivo* like state as possible. By manufacturing an optimised environment for hepatocytes we can address the need for appropriate *in vitro* models and the shortage of treatment options and donor livers for patients. By combining a valuable and underused resource such as human liver tissue with the reproducibility of polymeric scaffold manufacture we create a platform that can produce consistent, clinically translatable scaffolds for liver cell survival and function.

An electrospun fibre was chosen as the basis of these scaffolds as their fibrous nature mimics the morphology of native fibrillary collagen I in the liver extracellular matrix. PLA was used for this study because of its history of use in medical devices due to its biodegradation profile and compatibility with cellular environments. It is of note that the fibre diameter of each scaffold varied between proteins despite being electrospun under identical parameters. This method was employed to ensure the proteins were exposed to the same levels of electrical charge, solvent concentration and spinning time. The influence of fibre size when culturing upon electrospun scaffolds is yet to be fully elucidated, with evidence that variations between 0.3–1.3 µm do not influence cell behaviour^42,43^.

The livers used in this study are an underutilised resource. The donors are approved for transplant, but for varying reasons the liver may not be taken for transplant. In this situation, approved researchers are contacted and offered the tissue. Were we not to take the livers, they would be disposed of as clinical waste. This platform provides a method of using these livers to create niche, biologically active microenvironments for hepatocytes without the concerns that come with the more commonly used animal sourced tissue. Equally, the decellularization process developed as part of this work is effective and importantly, does not rely upon an intact vasculature for efficient decellularization unlike other successful work. This means that traumatically injured livers which are beyond surgical repair, those with occluded vasculature or partial lobes of liver. While the field of whole organ decellularization is a promising avenue for tissue engineers, it remains that the field is hampered by a lack of suitable whole organs. This method circumvents the need for whole organs. The platform vastly enlarges the pool of tissue available for this avenue of research and makes use of a precious resource donated by bereaved families which would otherwise go to waste when unsuitable for transplant.

Our results indicate that hLECM has an influence on hepatocytes which cannot be recapitulated by individual ECM components, confirming that there is a complex and not fully understood relationship between cells and the native extracellular matrix. We have demonstrated that this method is robust, reproducible and that by harnessing ECM protein in conjunction with 3D scaffolding technologies we produce a bioactive scaffold, which significantly alters the behaviour of liver cells.

ECM products are in clinical use, with products such as SIS, ALLO- PATCH HD^®^, MatriStem^®^, and Tutoplast^®^ all being used in regenerative medicine for the benefit of patients. Equally, our decellularization method completely removed the potentially immunogenic DNA from the ECM, allaying translational concerns.

To assess scaffold performance we used THLE-3 cells, a non-tumorigenic line derived from the left lobe of a normal adult human liver^44^. These cells were chosen because they are not cancer cells, however are immortalised and have been used in recent studies to represent non-tumourigenic hepatocytes^45–48^. While studies have debated the presence of CYP activity in THLE cells^41^, we propose that their non-tumourogenic growth pattern represented a test of scaffold biocompatibility with ‘normal’ hepatocytes, rather than with cancer derived cells. We assessed their adherence, growth and behaviour at 5, 10 and 16 days post-replating when cultured *in vitro* on the scaffolds containing hFN, hRL521, hBTC1 and hLECM versus scaffolds containing no protein; polymer only. We analysed cell attachment and viability, and gene expression of both liver function genes and ECM genes at both 5, 10 and 16 day time points. Additionally, we validated the retention of ECM proteins in the scaffolds through the electrospinning process and the effective decellularization of the human liver ECM.

This work has resulted in a robust platform for the production of blended protein:polymer scaffolds and a reproducible method of liver decellularization which does not rely on obtaining an entire organ. We have created bioactive scaffold for liver tissue engineering which influence the behaviour of THLE-3 human hepatocytes. There is vast potential for the application of these scaffolds and methodology in the field of liver tissue engineering and the wider tissue engineering community however further work is required to analyse the scaffolds influence on the cells and further improve the translatability of this work. While cell lines such as THLE-3s are undisputedly a highly valuable research resource, criticism of cell lines behaviour *in vitro* and the translatability of such results abounds within the scientific community^44,49^. It is important to undertake future work using primary or stem cell-derived hepatocytes^50–54^ and incorporate other important stimuli such as fluid flow to combat such criticism^55–57^. Furthermore, while the parenchymal hepatocytes make up more than 70% of the cellular mass, they do not exist in isolation and non-parenchymal and immune cells play an essential role in the *in vivo* liver; future studies should look to consider co-culture^58–60^.

We the researchers acknowledge the value of proteomic and functional assays (such as enzyme-linked immunosorbent assays) in analysing the function of the hepatocytes in any future studies. At this juncture these tests were deemed unnecessary considering the critiques and heterogeneous behaviour of cell lines^61–63^ and focus of this work on the development of a novel biomaterial for liver tissue engineering. Additionally, while every care was taken to ensure the complete removal of decellularization agents in the manufacture of these scaffold, this should be validated to ensure further translatability of this work, considering the deleterious effects of remnant detergent on cells^16,64,65^.

While such concerns are valid, this work clearly demonstrates the potential of blended ECM scaffolds for liver tissue engineering, utilising an underused and valuable resource of human liver tissue rejected for transplant and provides a robust initial platform for further research.

This work established a robust platform for the production of reproducible blended protein:polymer scaffolds for liver tissue engineering by combining human liver ECM with electrospun polymer manufacturing technologies. To do so, we created a consistent and effective method of decellularizing the tissue using a pressurised flow device. We solubilized the ECM and incorporated it directly into the fibres via the electrospinning solution. We made additional scaffolds for comparison containing hFN, hBTC1 and hRL521. These were all compared to a scaffold manufactured with no protein. The protein:polymer scaffolds were seeded with a liver cell line to assess their biological influence. The work was validated using robust methods such as Q-PCR, mechanical quantification, and SEM. Liver ECM containing protein:polymer scaffolds exert a significant positive influence on the gene expression profile, albumin production, attachment, and survival of liver cells.

Our results demonstrate great promise as a method of incorporating liver ECM and other proteins directly into a scaffold environment and of making impactful use of a valuable tissue resource which would otherwise be wasted. These scaffolds show great potential not only for the future of liver tissue engineering and patient treatment, but are easily adaptable for other organs and tissues. Additionally, they are a useful tool for the development of 3D liver cell platforms, which can be used for *in vivo* cell analysis and novel therapeutics research.

## Materials and Methods

### Ethics and governance

All human tissue used in this study was provided by NHS Organ Donation and Transplant and NHS Blood and Transplant. No organs/tissues were procured from prisoners. Ethical approval was granted for the project from the North of Scotland Research Ethics Committee, ref 16/NS/0083. Informed consent for organ donation for research purposes was obtained in accordance with the Helsinki Declaration.

### Decellularization

**Figure 8:**
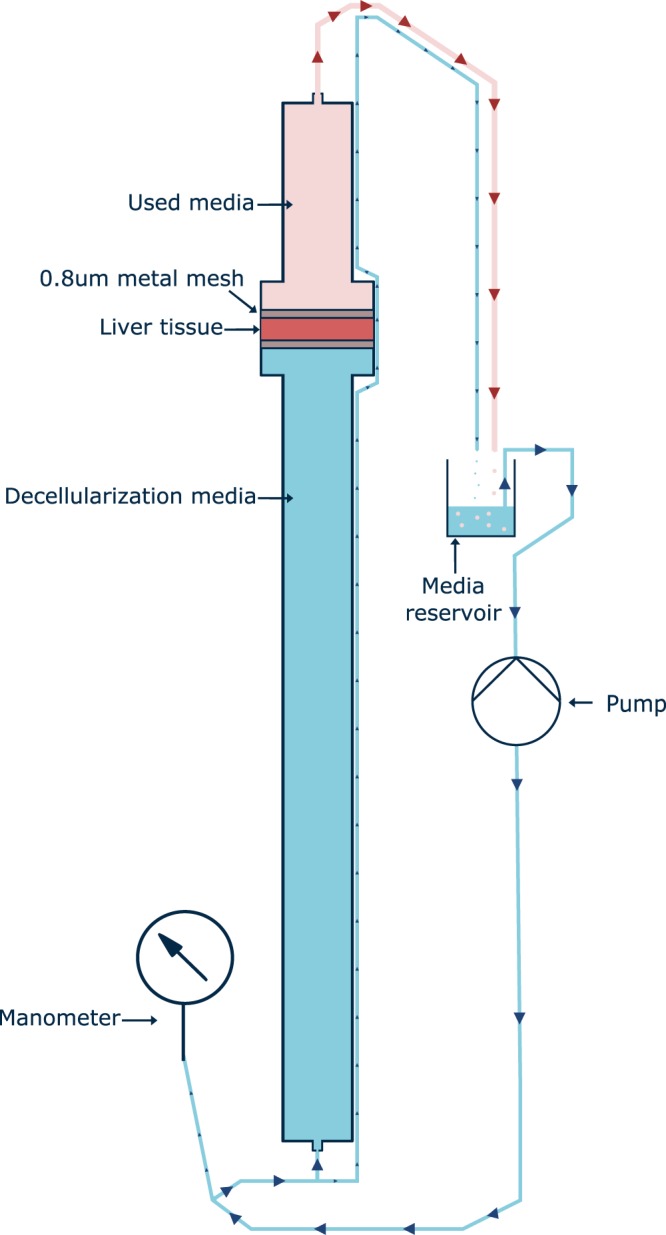
Decellularization device. Schematic of decellularization device which maintains pressurized flow to effectively decellularize tissue biopsies.

Decellularization of human liver tissue was performed at room temperature (19–22 °C) in a custom-made perfusion decellularization system (Fig. 8). Tissue was sliced into 3 mm thick sections and 35 mm diameter punches resected from the sections.

Tissue discs were placed in the decellularization device and secured between 70 µm stainless steel filtration mesh. A peristaltic pump (Watson Marlow 302 fixed speed pump) provided a flow rate of 200 ml/min. Pressure was adjusted to 24 mmHg using plasson pipes.

Tissue was subjected to an initial 4 hours of decellularization using 1 L of 0.5% sodium dodecyl sulphate (Sigma) in MilliQ H_2_O with 0.1% 100U/ml penicillin, 100 µg/ml streptomycin, 0.25 µg/ml Fungizone^®^ (amphotericin B) Anti-Anti solution (Gibco). After 4 hours, the decellularization solution was exchanged for fresh solution and the system ran overnight under the same conditions. The tissue was then washed with MilliQ H_2_O with 0.1% 100U/ml penicillin, 100 µg/ml streptomycin, 0.25 µg/ml Fungizone^®^ (amphotericin B) Anti-Anti solution (Gibco) for 4 hours and stored in sterile containers at −20 °C until use.

### Preparation of hLECM

Decellularization was confirmed using the Quant-IT^™^ Picogreen^®^ dsDNA assay kit (Life Technologies^™^), performed according to manufacturer’s instructions. The ECM was lyophilized in a FreeZone^®^ 4.5 freeze-drier (Labconco^®^) before milling in a PM100^®^ planetary ball mill (Retsch^®^).

### Preparation of proteins for electrospinning

The powdered hLECM was solubilized in 0.25 M acetic acid (Acros Organics) at a concentration of 25 µg per ml. Bornstein and Traub Type I collagen (Sigma Type VIII), powder from human placenta (hBTC1), human recombinant laminin 521 (hRL521) (Biolamina), and human plasma fibronectin (hFN) (Merck) were all solubilized and incorporated into the electrospinning solutions using the same methods. A control scaffold consisting of polymer only in the 9:1 HFIP:0.25 M acetic acid was also incorporated into the study.

### Preparation of electrospinning solutions

Methods henceforth were described previously in Grant *et al*.^7^ and Grant *et al*.^66^. Briefly, a 22% wt/vol solution of poly-L-lactic acid (Goodman) and 9 ml hexafluoroisopropanol (Manchester Organics) was dissolved overnight at room temperature with agitation. 1 ml of 0.25 M acetic acid (Acros Organics) containing 25 µg protein was added to the 22% poly-L-lactic acid:HFIP solution and combined at room temperature under agitation for 1 hour.

### Electrospinning

Electrospinning was performed using a syringe pump EP-H11 (Harvard Apparatus) and an EC-DIG electrospinning system (IME technologies) via a 27G bore needle under the parameters noted in Table 3; Mandrels were coated in non-stick aluminium foil to collect the electrospun fibres. Fibre sheets were allowed to dry overnight in a fume hood and stored at 4 °C until use. Average fibre size of each scaffold type was as calculated using ImageJ plugin ‘DiameterJ’^67^ and is reported in Table 4.

**Table 3:** Electrospinning parameters.

Volume per hour	Total volume	Mandrel:needle distance	Positive charge	Negative charge	Mandrel rotation	Needle movement
2.5 ml	7.5 ml	23 cm	16 kV	−3 kV	300 rpm	100 mm/s

**Table 4:** Fibre sizes.

Scaffold	Polymer only	hBTC1	hFN	hRL521	ECM
Average fibre Size (µm)	1.82	1.60	1.72	1.17	2.01

### Scaffold Preparation

10 mm scaffold discs were cut using a biopsy punch. Scaffolds were submerged in 30% isopropyl alcohol for 10 minutes, then subsequently rinsed three times in phosphate buffered saline for 15 minutes each. Scaffolds were submerged in an antibiotic/antimycotic treatment solution of Dulbeccos Minimal Essential Media supplemented with 100U/ml penicillin, 100 µg/ml streptomycin, 0.25 µg/ml Fungizone^®^ (amphotericin B) Anti-Anti solution (Gibco) for 1 hour.

### Cell Seeding and Culture

THLE-3 cells were trypsinized using standard methods from tissue culture flasks and counted using the trypan blue exclusion method. 1 × 10^5^ cells at passage 14 were suspended in 100 µl of complete media and seeded directly on to the scaffolds. The cells were allowed adhere for 2 hours, then an additional 400 µl of complete media was added.

Media was changed after 24 hours and changed every 48 hours subsequently. This functional layer (FL) of cells was cultured using standard methods for 5, 10 or 16 days at 37 °C and 5% CO_2_ in a humidified incubator.

### Live/Dead^®^ Viability/Cytotoxicity assay

Cell/scaffold constructs were incubated with 10 µm calcein and 2 µm ethidium homodimer-1 (Ethd-1) for 30 minutes as part of the two colour live/dead assay (Molecular Probes). The scaffolds were rinsed three times in CaCl_2_/MgCl_2_ free PBS to remove excess dye and placed onto a standard microscope slide with a 25 mm glass coverslip (VWR). All images were captured using a Zeiss Axio Imager fluorescent microscope (COIL, University of Edinburgh) at 40x magnification and post processed using ImageJ.

### CellTiter-Blue^®^ Cell viability assay

The assay was performed according to manufacturer’s instruction (Promega). For each condition group, minimum n = 5. Importantly, cell/scaffolds constructs were moved into fresh 48 well plates to prevent reading activity from tissue culture plastic bound cells. Measurements were read in a Modulus^™^ II microplate reader at an excitation wavelength of 525 nm and emission wavelength of 580–640 nm and reported as fluorescence.

### Albumin quantification

The bromocresol green (BCG) albumin assay (Sigma) was performed according to manufacturer’s instructions and was used to quantify serum albumin produced by the FL over 24 hours at 5, 10 and 16 day time points. Results were read at an absorbance of 620 nm in a Modulus™ II microplate reader. For each condition group, minimum n = 5.

### Picogreen^®^ DNA quantification

The Quant-IT Picogreen^®^ dsDNA assay kit (Life Technologies^™^) was performed according to manufacturer instructions to establish the efficiency of the decellularization method and to estimate cell adherence and growth on the cell/scaffold constructs. Fluorescent intensity measurements were read in a Modulus^™^ II microplate reader (minimum n = 5) at an excitation wavelength of 480 nm and emission wavelength of 510–570 nm. A standard λ dsDNA curve of graded known concentrations was used to calibrate fluorescence intensity vs dsDNA concentration.

### Immunohistochemistry

The samples were rinsed three times in PBS (Gibco) for 15 minutes each, then fixed in 4% v/v formalin buffered in saline for 1 hour at room temperature. Immunohistochemical staining was undertaken using antibodies for Collagen I (Stratech), Laminin (Stratech) and Fibronectin (Sigma). All images were captured using a Zeiss Axio Imager system (Centre Optical Instrumentation Laboratory, University of Edinburgh) at 40x magnification and post processed using ImageJ.

### Scanning Electron Microscopy

SEM was used to characterise the scaffold architecture. Samples were rinsed three times in PBS for 15 minutes each, then fixed in 2.5% v/v glutaraldehyde (Fisher Scientific) in 0.1 M phosphate buffer (PB) (pH 7.4) at 4 °C overnight. They were then rinsed three times in 0.1 M PB before being post-fixed in 1% v/v osmium tetroxide (Electron Microscopy Supplies) buffered with 0.1 M PB. Samples were again rinsed three times in 0.1 M PB and dehydrated through an ethanol gradient (30–100%). They were dried by placing them in hexamethyldisilazane (HMDS, Sigma) which was allowed to evaporate off at room temperature overnight. We mounted the samples onto SEM chucks using double sided carbon tape and coated them with a thin layer of gold and palladium alloy (Polaron Sputtercoater).

All images were captured at 5 kV using a Hitach S-4700 SEM (BioSEM, University of Edinburgh).

### Mechanical testing

Tensile testing was undertaken to establish the dynamic properties of scaffolds and hLECM using the Instron 3367 dual column universal testing system with Bluehill 3 software. The system was fitted with Instron biopulse submersible pneumatic side action grips and a 50 N load cell. A gauge length of 20 mm and an extension rate of 20 mm/min were used for all tensile tests. Analyses was conducted using the incremental modulus method as previously described^68,69^.

Six samples of each scaffold with a width of 10 mm and a gauge length of 80 mm were obtained from each sheet. Samples were fixed to ‘C’ shaped card templates to allow consistent set up during tensile testing. Samples were tested until failure. N = 6.

### Gene expression analysis

All kits were used according to manufacturer’s instructions. RNA was extracted from FL cells using standard Trizol (Fisher Scientific) methods and purified using Qiagen’s RNeasy spin column system. cDNA was synthesised using the Promega ImProm-II^™^ Reverse Transcription System.

Quantitative real-time polymerase chain reaction (qRT-PCR) was performed using the LightCycler^®^ 480 Instrument II (Roche Life Science) and Sensifast^™^ SYBR^®^ High-ROX (Bioline) system. Results were normalized to THLE-3s of the same passage number grown on tissue culture plastic and compared to the housekeeping gene Glyceraldehyde-3-Phosphate Dehydrogenase (GAPDH). Analysis was performed using the 2–[delta][delta] Ct method^70,71^, minimum n = 5. Albumin (Alb), Cytochrome P450 Family 1 Subfamily A Polypeptide 1 (Cyp1A1), Cytochrome P450 Family 1 Subfamily A Polypeptide 2 (Cyp1A2), Cytochrome P450 Family 3 Subfamily A Polypeptide 4 (Cyp3A4), Collagen Type I alpha 1 (Col1A1), Collagen Type 4 alpha 1 (Col4A1) and Fibronectin Type 1 (FN1), alpha-1 antitrypsin (AAT) and hepatocyte nuclear factor (HNF) were investigated, forward and reverse primers (Sigma) are detailed in Supplementary Table 1.

### Statistical analysis

One-way ANOVAs with Games-Howell and Tukey post-hoc testing was performed using Minitab 18 Statistical Software. Multiple comparisons tests were used following the Ryan Joiner test for normality and Bartlett’s test for the homogeneity of variances. The Tukey post hoc test was used where Bartlett’s test result is not significantly different i.e. the null hypothesis of population variances being equal is not rejected. The Games-Howell test does not assume equal variances and sample sizes and was performed on the ranked variables similar to other nonparametric tests. The Games-Howell post hoc test is used where Bartlett’s test result is significantly different i.e. the null hypothesis of population variances being equal is rejected. Error bars indicate standard deviation. A minimum of n = 3 and max of n = 6 was used for all analysis. *p value < 0.05, **p value < 0.01, ***p value < 0.001.

### Data sharing

All data generated or analysed during this study are included in this published article (and its Supplementary Information files).

## Supplementary information

Supplementary Data
