# Supplementary material for: Blended electrospinning with human liver extracellular matrix for engineering new hepatic microenvironments
Source: Sci Rep. 2019 Apr 18;9:6293. doi: 10.1038/s41598-019-42627-7 (PMC6472345; doi:10.1038/s41598-019-42627-7)
Supplement: Supplementary file 1 — Supplementary Data [file 41598_2019_42627_MOESM1_ESM.pdf]

Blended electrospinning with human liver extracellular matrix for engineering new hepatic microenvironments – Supplementary data

Rhiannon Grant[1], Dr John Hallett[2], Prof Stuart Forbes[2], Prof David Hay[2], Dr Anthony Callanan[1*]

1. Institute for Bioengineering, School of Engineering, University of Edinburgh, Scotland, UK

2. Scottish Centre for Regenerative Medicine, University of Edinburgh, Scotland, UK

Corresponding author: anthony.callanan@ed.ac.uk

Supplementary Figure 1; AAT and HNF gene expression

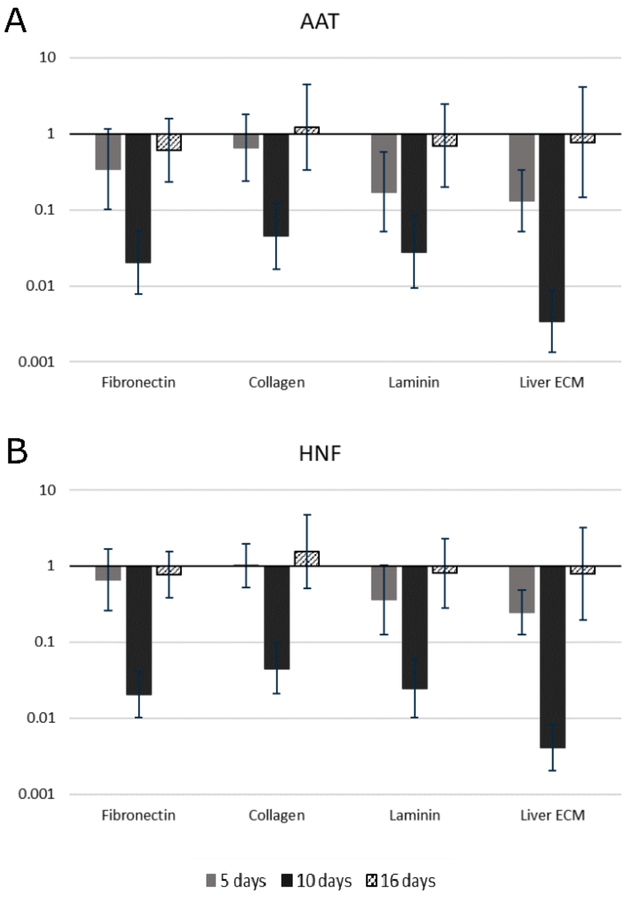

*Supplementary figure 1; Quantitative analysis of gene expression was undertaken on the functional cell layer at five, ten and sixteen days of culture, compared to that of the same culture periods grown on polymer only scaffolds. mRNA levels of Alpha-1 aminotransferase (A) and Hepatocyte nuclear factor (B) are represented as fold difference relative to tissue culture plastic controls and relative to the housekeeping gene GAPDH. One-way ANOVA with Games Howell and Tukey post hoc testing and minimum n = 5. * = p <0.05 ** = p<0.01, *** = p<0.001. Error bars = SD.*

Supplementary Table 1. RT-PCR primers

Gene	Sequence
**Albumin (Alb)**	For - CCTGTTGCCAAAGCTCGATG<br>Rev – GAAATCTCTGGCTCAGGCGA
**Cytochrome P450 Family 1 Subfamily A Polypeptide 1 (Cyp1A1)**	For - AATTTCGGGGAGGTGGTTGG<br>Rev – GATGTGGCCCTTCTCAAAGGT
**Cytochrome P450 Family 1 Subfamily A Polypeptide 2 (Cyp1A2)**	For - CTTCGCTACCTGCCTAACCC<br>Rev – GTCCCGGACACTGTTCTTGT
**Cytochrome P450 Family 3 Subfamily A Polypeptide 4 (Cyp3A4)**	For- TTTTTGGATCCATTCTTTCTCTCAA<br>Rev- TCCACTCGGTGCTTTTGTGT
**Collagen Type I alpha 1 (Col1A1)**	For - GGACACAGAGGTTTCAGTGGT<br>Rev – GCACCATCATTTCCACGAGC
**Collagen Type 4 alpha 1 (Col4A1)**	For - GACCCCCGGGAGAAATAGGT<br>Rev – TTTGAAAAAGCAATGGCACTCC
**Fibronectin Type 1 (FN1)**	For - GAACAAACACTAATGTTAATTGCCC<br>Rev – TCTTGGCAGAGAGACATGCTT
**Alpha-1 aminotransferase (AAT)**	For – GATGCTGCCCAGAAGACAGA<br>Rev - GGAGTTCCTGGAAGCCTTCA
**Hepatocyte nuclear factor (HNF)**	For – TCGTTGAGTGGGCCAAGTAC<br>Rev - TGTCATCGATCTGCAGCTCC
**Glyceraldehyde-3-Phosphate Dehydrogenase (GAPDH)**	For – GTCTCCTCTGACTTCAACAG<br>Rev – GTTGTCATACCAGGAAATGAG
